# Variation of Tumor Volume During Moderate Hypo-Fractionated Stereotactic Body Radiation Therapy for Lung Cancer

**DOI:** 10.7759/cureus.17743

**Published:** 2021-09-05

**Authors:** Keiichiro Nishimura, Shogo Hatanaka, Nobuko Utsumi, Takafumi Yamano, Munefumi Shimbo, Takeo Takahashi

**Affiliations:** 1 Department of Radiation Oncology, Saitama Medical Center, Saitama Medical University, Kawagoe, JPN; 2 Department of Radiation Therapy, JCHO Tokyo Shinjuku Medical Center, Tokyo, JPN; 3 Department of Radiation Oncology, Saitama Medical Center, Saitama Medical University, Saitama, JPN

**Keywords:** stereotactic body radiation therapy (sbrt), moderate hypo-fractionated sbrt, lung cancer, variation of tumor volume, maximum standardized uptake values

## Abstract

Aim

To investigate the variation of tumor volume during moderate hypo-fractionated stereotactic body radiation therapy (SBRT).

Patients and Methods

Twenty patients, who received SBRT at our institution, were included in the analysis. A prescribed dose was 56 Gy at iso-center in seven fractions. Tumor volumes before and during SBRT were calculated. In order to investigate factors affecting the variation of tumor volume in RT 2 (after first irradiation) and RT 7 (after last irradiation), various parameters were verified by the Mann-Whitney *U *test.

Results

With regard to the low maximum standardized uptake values (SUVmax) group, transient increase of tumor volume was found in RT 2, and tumor volume reduction was hardly found in RT 7. With regard to the high SUVmax group, a transient increase was not found, and a definite reduction was found in the treatment course.

Conclusion

Accurate prediction of tumor volume variation is required for more accurate treatment, such as adaptive radiation therapy.

## Introduction

Stereotactic body radiation therapy (SBRT) is a treatment technique for irradiation to localized cancer in a trunk of a body accurately. With this technique, total doses of radiation are divided into large doses, and radiation treatment is given over a shorter period compared to conventional radiation therapy. At present, the SBRT technique has been used widely for the treatment of stage I non-small cell lung cancer [1]. In Japan, SBRT with 48 Gy in four fractions is implemented at many institutions according to Japan Clinical Oncology Group 0403 protocol [2, 3]. Ohnishi et al. reported that the 5-years survival rate was 72%, and the local control rate was 87-97％ by using SBRT for stage I non-small cell lung cancer in a multi-institutional study under the condition that biological effective doses (BED) were ≥100 Gy (α/β = 10 Gy) [4]. Similarly, by using SBRT with 48 Gy in four fractions, Nagata et al. reported that the 3-years survival rate was 83% [5]. However, both reports investigated only for peripheral lung cancer, and central lung cancer was not included. In a previous report, grade 5 adverse events were found after SBRT in patients of central lung cancer with 48 Gy in 4 fractions [6]. Therefore, regarding central lung cancer, moderate hypo-fractionated SBRT (e.g., 56 Gy in seven fractions) might be useful for the reduction of late effect in normal tissues.

In radiation therapy, the accurate decision of gross tumor volume (GTV) and target volume is important; thus, it is required to consider the variation of tumor volume during the course of treatment. Recently, even for SBRT with a short period of treatment, variation of tumor volume during the course of treatment has been investigated in several reports [7-9]. Regarding SBRT with 48-52 Gy in four fractions, Tatekawa et al. reported ≥10 % increase in tumor volume was detected in 16 of 50 cases. For that reason, considering the tumor volume is important in order to perform a more accurate treatment, such as adaptive radiation therapy [7]. However, a mechanism of this transient increase during SBRT is not clear. Additionally, no report has verified variation of tumor volume during moderate hypo-fractionated SBRT. Therefore, we investigated the variation of tumor volume during moderate hypo-fractionated SBRT in this study.

## Materials and methods

Table 1 shows patient information. From April 2015 to December 2016, 20 patients (21 plans), who received SBRT at our institution, were included in the analysis. All procedures were approved by the Ethical Committee of our institution.

**Table 1:** Patient information

Gender	Male	Female
	14	6
Age	rage	mean
	67 - 88	76
Operation history of lung	yes	no
	6	15
Pathology		
adenocarcinoma	10	
squamous cell carcinoma	1	
non-small cell carcinoma	1	
unknown	5	
metastases^※^	4	
^※^primary lesion (rectum 1, esophagus 1, renal cell 1, ureteral 1)		
Tumor site	distal side	central side
	20	1
	left lobe	right lobe
	9	12
	superior side	inferior side
	15	6
	anterior side	posterior side
	12	9
Tumor size	range	mean
	7 - 39mm	22.8 mm
Destruction of alveolar wall around the tumor	yes	no
	9	12

2-1. Treatment planning

**Figure 1:**
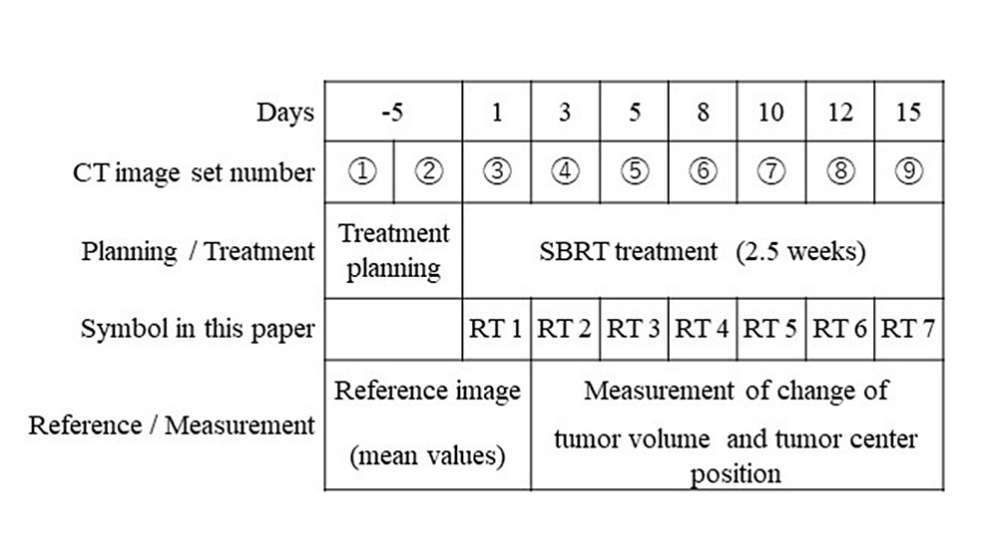
Schedule of Stereotactic Body Radiotherapy

SFORM ESN-1800 (Engineering system) was used for a patient fixture. The breathing of the patient was suppressed by chest compression. computed tomography (CT) images for dose calculation were acquired by GE Optima CT580 (GE Medical Systems, Chicago, Illinois) with a slow scan method in four seconds per rotation under the condition of free breathing. The slice thickness was 1.25 mm, tube voltage was 120 kV, and automatic exposure control was used to regulate tube current (36.6 mA to 500 mA). XiO (Elekta, Stockholm, Sweden) was used as the radiation treatment planning system (RTPS). The linear accelerator Clinac21EX (Varian Medical Systems, Helsinki, Finland) with a 6 MV X-ray was used as a radiation source. Created plans contained non-coplanar 8-10 fields. The GTV and internal target volume (ITV) were determined based on CT images. The planning target volume (PTV) was defined by the ITV with a seven mm margin. The radiation fields were optimized to fit the PTV with a multi-leaf collimator (MLC) margin of five mm. A prescribed dose was 56 Gy at iso-center in seven fractions (three fractions per week). Figure 1 shows a summary of the treatment course.

2-2. Variation of tumor volume during SBRT

**Figure 2:**
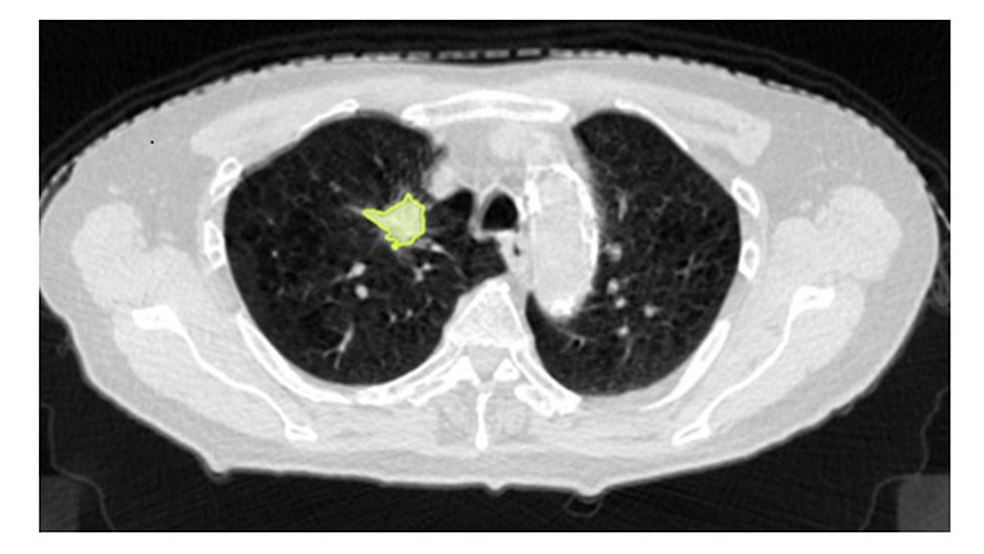
Contour extraction for SBRT An automatic contouring function installed in Velocity software was used for acquiring tumor volume in this study. A threshold value of a CT value was -250 HU. In addition, one experienced radiation oncologist excluded blood vessels and chest walls manually.

An automatic contouring function installed in Velocity 3.2.1 (Varian Medical Systems, Helsinki, Finland) was used for acquiring tumor volume in this study. A threshold value of a CT value was -250 Hounsfield units (HU) [7]. In addition, one experienced radiation oncologist excluded blood vessels and chest walls manually. Figure 2 shows an example of a tumor contour. Ratios of reference tumor volume (TVR) and tumor volume during SBRT (TV2-TV7) were calculated (TVX/TVR). An average value of tumor volume which was acquired by three CT images (1st planning CT, 2nd planning CT, and RT 1 CT images) was used as TVR (see Figure 1).

2-3. Statistical analysis

In order to investigate factors affecting the variation of tumor volume in RT 2 (after first irradiation) and RT 7 (after last irradiation), the following parameters were verified by the Mann-Whitney U test. Gender, Age (< 70 or ≥70, and < average:76 or ≥76), Presence of lung surgery anamnesis, tumor volume before SBRT (<average：4.4 cm^3^ or ≥ 4.4 cm^3^), lung volume (< average：2875 cm^3^ or ≥ 2875 cm^3^), Presence of alveolar wall destruction near tumors, tumor locations (peripheral or central, left or right lobe, head or foot side, and ventral or dorsal side), and maximum standardized uptake values (SUVmax) acquired by 18F-FDG-Positron Emission Tomography (PET) images. FDG-PET / CT is widely used and the most reliable modality to evaluate the tumor metabolic activity using the glucose metabolism independent of morphological change. Although various parameters in FDG-PET/CT have been reported, SUVmax is the most common and reliable parameter to evaluate tumor metabolic activity. Since this study is a retrospective study, the constantly measured and reliable SUVmax was used as a biomarker in this study. The tumor locations of head-foot sides were distinguished by halfway lines between lung apexes and bottoms as boundaries. The tumor locations of ventral-dorsal sides were distinguished by vertebral leading edges as boundaries.

## Results

**Figure 3:**
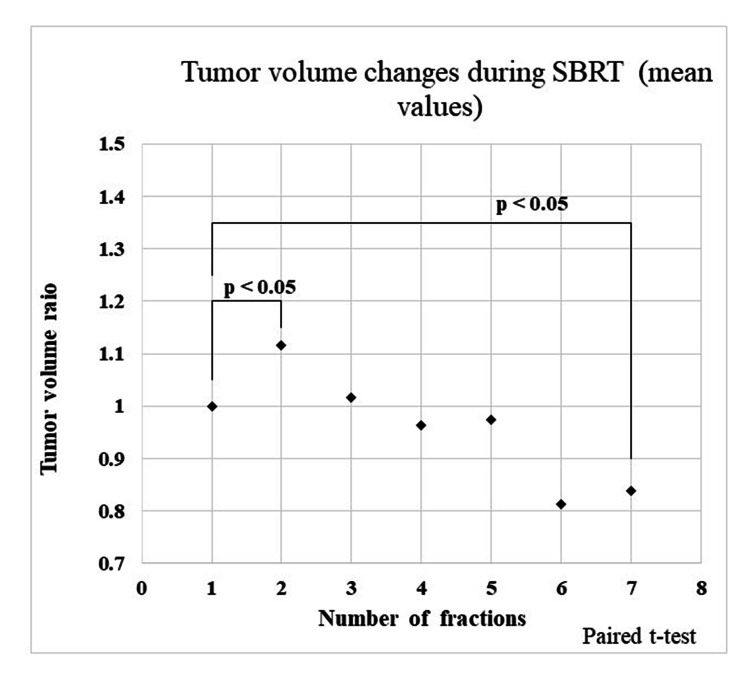
Tumor volume changes during SBRT (mean values) A definite increase was found in TV2, on the other hand, a definite decrease was found in TV7. SBRT - stereotactic body radiotherapy

**Figure 4:**
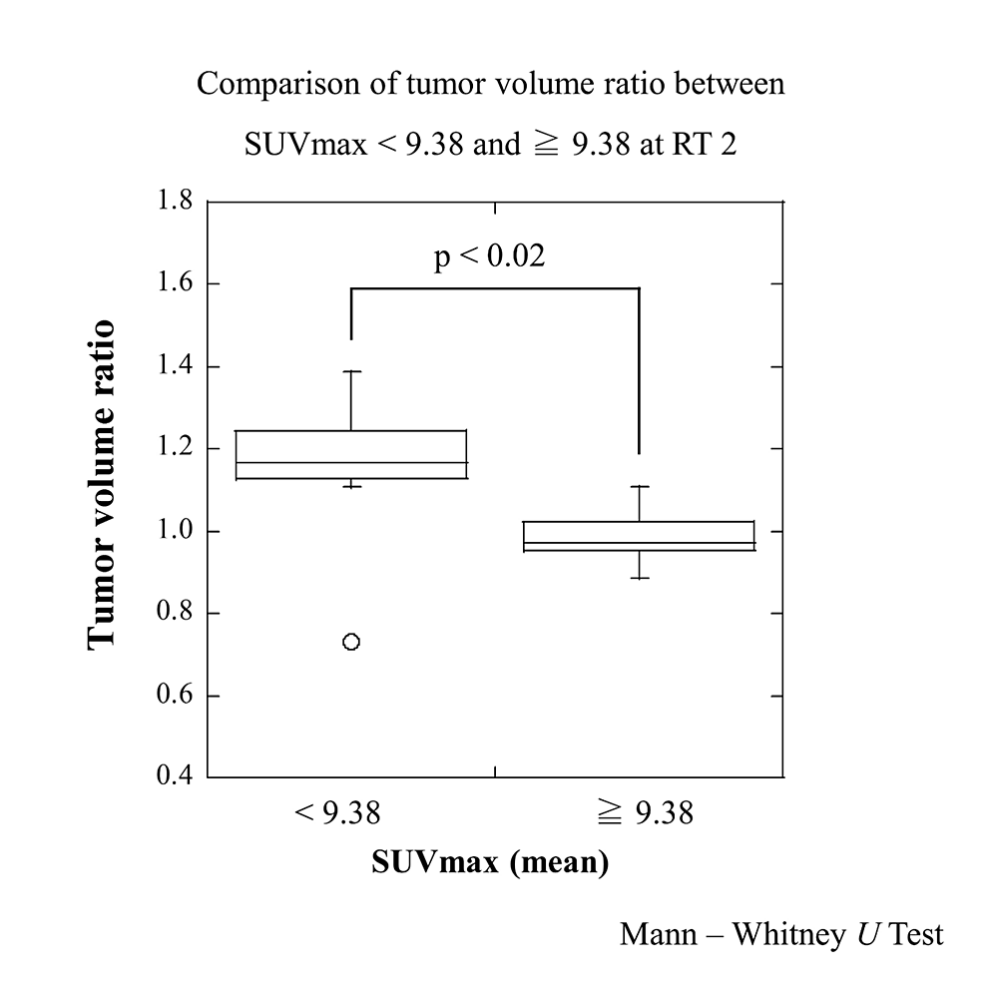
Comparison of tumor volume ratio between SUVmax < 9.38 and ≥ 9.38 at RT2 A TV2/TVR mean value of SUVmax in the < average group was larger than a TV2/TVR mean value of SUVmax in the ≥ average group (p < 0.02). SUVmax - maximum standardized uptake values

**Figure 5:**
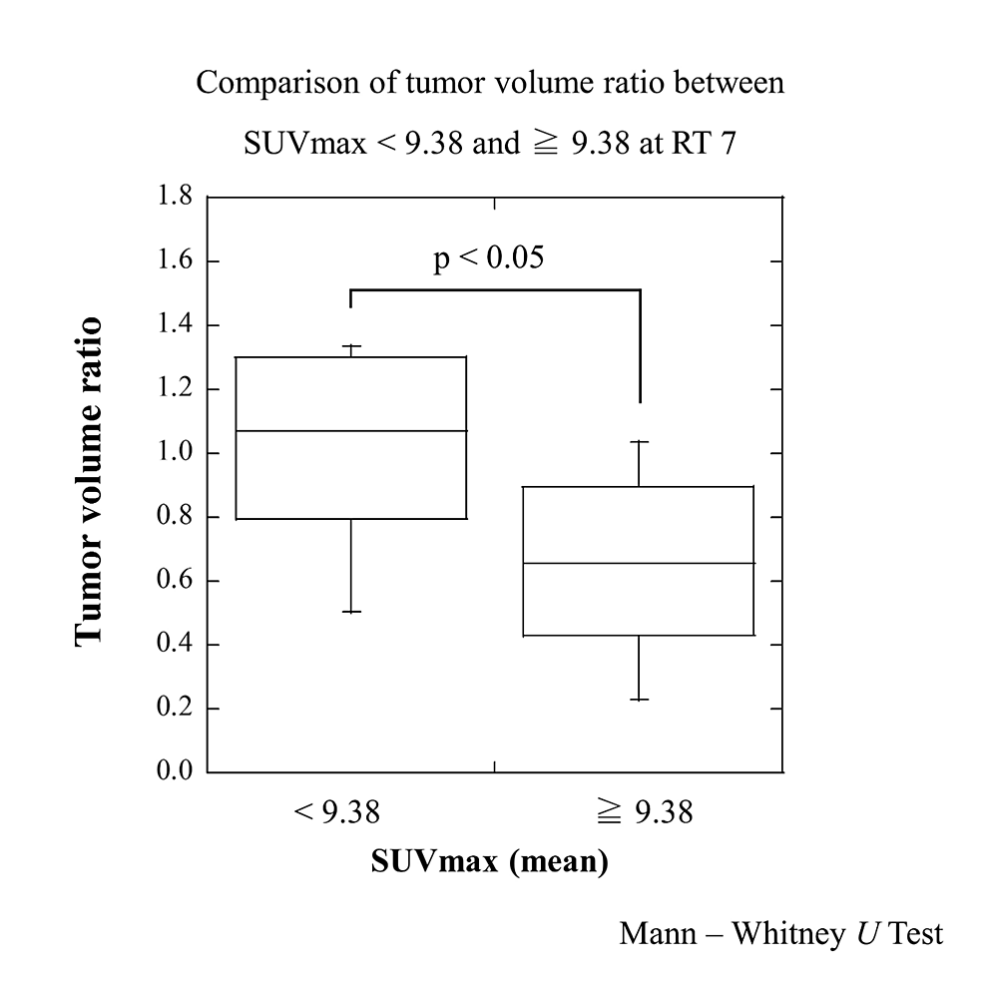
Comparison of tumor volume ratio between SUVmax < 9.38 and ≥ 9.38 at RT7. A TV7/TVR mean value of SUVmax in the < average group was larger than a TV7/TVR mean value of SUVmax in the ≥ average group (p < 0.05). SUVmax - maximum standardized uptake values

**Figure 6:**
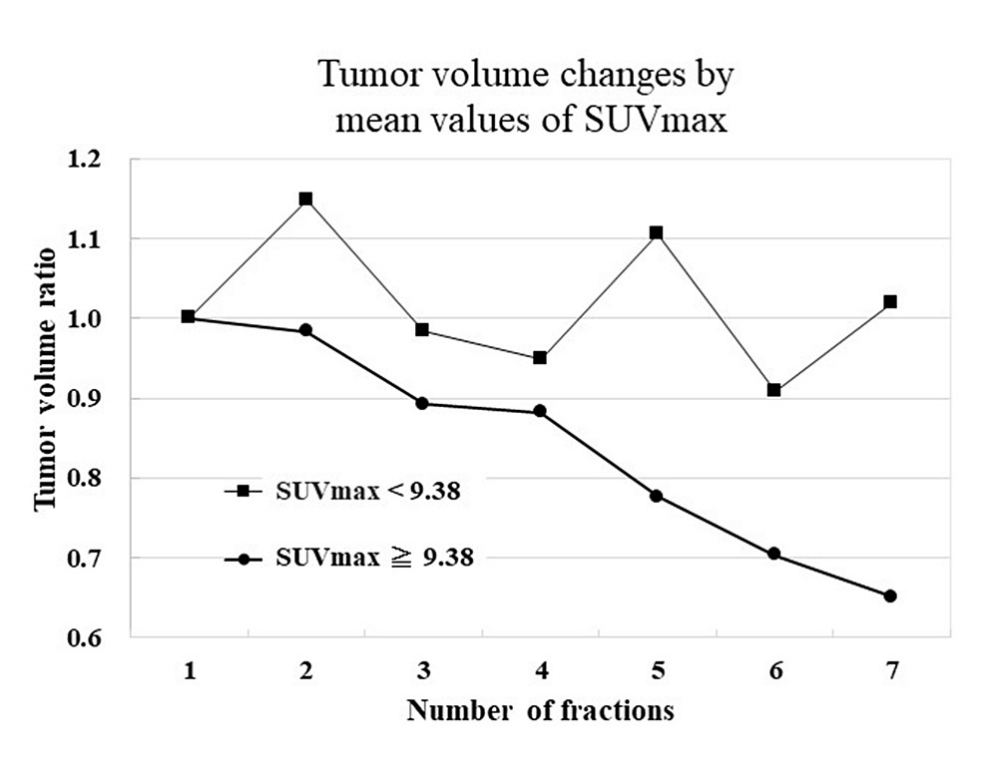
Tumor volume changes by mean values of SUVmax With regard to the values of SUVmax in the < average group, a transient increase of tumor volume was found in RT 2, and tumor reduction was hardly found in the latter half of the treatment course. With regard to the values of SUVmax in the ≥ average group, a transient increase of tumor volume was not found, and definite tumor reduction was found in the latter half of the treatment course. SUVmax - maximum standardized uptake values

Figure 3 shows ratios of reference tumor volume and tumor volume during SBRT and results of paired t-test. A definite increase was found in TV2; on the other hand, a definite decrease was found in TV7. Results of statistical analysis for variation of tumor volume (TV2/TVR and TV7/TVR) were shown in Table 2. Regarding gender, age, presence of lung surgery anamnesis, tumor volume before SBRT, lung volume, presence of alveolar wall destruction, and tumor location, we did not find a significant association between these and the variation of tumor volume. On the other hand, a TV2/TVR mean value of SUVmax in the < average group was larger than a TV2/TVR mean value of SUVmax in the ≥ average group (p < 0.02) as showed in Figure 4. Similarly, a TV7/TVR mean value of SUVmax in the < average group was larger than a TV7/TVR mean value of SUVmax in the ≥ average group (p < 0.05), as showed in Figure 5. Figure 6 shows a comparison of variation in TVX/TVR during SBRT between values of SUVmax in the ≥ average group and values of SUVmax in the ≥ average group. With regard to the values of SUVmax in the < average group, a transient increase of tumor volume was found in RT 2, and tumor reduction was hardly found in the latter half of the treatment course. With regard to the values of SUVmax in the ≥ average group, a transient increase of tumor volume was not found, and definite tumor reduction was found in the latter half of the treatment course.

**Table 2:** Results of statistical analysis for variation of tumor volume (TV2/TVR and TV7/TVR) N.S. - not significant

	Group		TV_2_/TV_R_	TV_7_/TV_R_
Gender	Male	Female	N.S.	N.S.
Operation history of lung	yes	no	N.S.	N.S.
Tumor volume	＜4.4㎝^3^	≧4.4㎝^3^	N.S.	N.S.
Lung volume	＜2875㎝^3^	≧2875㎝^3^	N.S.	N.S.
Destruction of alveolar wall around the tumor	yes	no	N.S.	N.S.
Tumor site	distal site	central site	N.S.	N.S.
	left lobe	right lobe	N.S.	N.S.
	superior side	inferior side	N.S.	N.S.
	anterior side	posterior side	N.S.	N.S.
SUVmax	＜9.38	≧9.38	p＜0.02	p＜0.05

## Discussion

Regarding chemo-radiotherapy with standard fractionated irradiation, which requires six weeks for treatment, it has been reported that reduction of tumor volume was found in 40-50% cases with non-small cell lung cancer [10,11] and was found in 65% cases with small cell lung cancer [10]. Variation of tumor volume during the treatment course had not been investigated until a while ago because the treatment period of SBRT was very short (about one week). However, recently Tatekawa K et al. [7] and Gunter T et al. [8] reported that transient increase of tumor volume during treatment course in SBRT with 4-5 fractions was found. Bhatt et al. had reported that a decrease of tumor volume during SBRT with five fractions was found [9]. In regard to this study, with moderate hypo-fractionated SBRT, which requires 2.5 weeks for treatment, a definite transient increase of tumor volume was found in RT 2. It is unlikely that the tumor growth is found during the short treatment course of SBRT using a large dose per fraction compared with conventional radiotherapy. Because it is thought that the time when the tumor increases transiently in RT2 of SBRT does not coincide with the time when repopulation of the tumor occurs. Therefore, it was considered that the transient increase of the tumor was not due to the growth of the tumor but due to the effect of irradiation on the interstitial component of the tumor, such as edema caused by large irradiation dose per fraction [7]. On the other hand, a definite reduction of tumor volume was found in RT 7. Regarding gender, age, presence of lung surgery anamnesis, tumor volume before SBRT, lung volume, presence of alveolar wall destruction, and tumor locations, we found no significant association between these and variation of tumor volume. Verifying the relationship between SUVmax value and variation of tumor volume, trends were different between a high SUVmax value group and a low SUVmax value group. A high proliferation rate for cells and a high growth rate for tissues result in increased radiosensitive. Investigation of the relationship between the expression of biomarkers related to tumor proliferation and radio-sensitivity had been implemented [12]. However, for radiotherapy of small lung cancer, the use of biomarkers obtained by tumor tissues was difficult. Therefore, SUVmax values obtained by 18F-FDG-PET/CT images were used for a biomarker in this study. It has been reported that 18F-FDG-PET/CT has high sensitivity (96.8%) and moderate specificity (77.8%) for the diagnosis of lung cancer [13]. SUVmax values and tumor proliferation were correlated [14, 15]. In a high SUVmax value group, tumor reduction was found in an early stage of the treatment course because tumor proliferation rates and radio-sensitivity might be high. On the other hand, in a low SUVmax value group, a transient increase of tumors was found in an early stage of the treatment course because of the influence of interstitial change such as edema caused by large radiation doses [7].

As a limitation, a slow-scan technique was used for the acquisition of CT images in this study. Regarding the accurate acquisition of moving tumor shapes, the slow scan is inferior to high-speed scan techniques such as 4D-CT [16, 17]. However, a slow scan can acquire tumor central position and a moving range accurately [16, 17]. Therefore, we expected that a slow scan CT could be used for the evaluation of relative tumor volume variation.

From now on, an increasing ratio of elderly patients with lung cancer is predicted. Sandhu AP et al. had reported that the 2-years disease-free survival rate was 77%, and the 2-years overall survival rate was 74% for elderly patients from 80 to 90 years old with stage I non-small cell lung cancer by using SBRT [18]. Additionally, they had reported that SBRT was a useful choice for the treatment of elderly patients with early non-small cell lung cancer [18]. Therefore, for patients who cannot undergo surgery with stage I-II non-small cell lung cancer, metastatic lung cancer, and liver cancer, SBRT might be a better choice for treatment [18, 19]. The utility and safety of SBRT with 4 fractions for peripheral lung cancer are established; however, an increase of adverse events is concerned for patients with central lung cancer. Haseltine JM et al. had investigated adverse events after SBRT with 48 Gy in four fractions - 50 Gy in five fractions for central lung cancer [20]. According to this report, when the distance from a bronchial tube to a tumor was < 1 cm, the frequency of adverse events of ≥ grade 3 was definitely increased [20]. On the other hand, in SBRT with an increased number of fractions, a decrease of serious adverse events was found [21, 22]. In the SBRT phase I trial (JROSG 10-1) for stage IA central non-small cell lung cancer, it had been reported that SBRT with 60 Gy in eight fractions was appropriate for stage IA central lung cancer from a perspective of adverse events [23]. Shibamoto et al. had reported that SBRT with 60 Gy / eight fractions / three fractions per week was useful for lung cancer ≥ 2 cm in size from a perspective of radiobiology [24]. Thus, the moderate hypo-fractionated SBRT of this study (56 Gy / seven fractions / three fractions per week) might also be useful for both elderly and central lung cancer patients. However, transient increase of tumor volume during SBRT might cause dose deficiency of edge parts of tumors. An accurate definition of targets and appropriate setting of PTV margins were required for effective SBRT. The SUVmax value might be useful for prediction of transient increase during SBRT.

## Conclusions

In this study, variation of tumor volume during moderate hypo-fractionated SBRT for non-small cell lung cancer and metastatic lung cancer was investigated. As a result, a transient increase in tumor volume was found in an early stage of the treatment course. Especially if SUVmax value was < 9, a transient increase during SBRT was likely to occur. Accurate prediction of tumor volume variation is required for more accurate treatment, such as adaptive radiation therapy.
